# Facial nerve in skullbase tumors: imaging and clinical relevance

**DOI:** 10.1186/s40001-023-01078-7

**Published:** 2023-03-14

**Authors:** Longping Yao, Baoyan Wang, Fengfei Lu, Xiaozheng He, Guohui Lu, Shizhong Zhang

**Affiliations:** 1 Department of Neurosurgery, Zhujiang Hospital, Southern Medical University, Guangzhou, 510282 China; 2 Department of Neurosurgery, First Affiliated Hospital of Nanchang University, Nanchang, China

**Keywords:** Facial nerve, Anatomy, Histology, Skullbase tumors, MR imaging, Vestibular schwannoma

## Abstract

Facial nerve, the 7th cranial nerve, is a mixed nerve composed of sensory and motor fibers, and its main branch is situated in the cerebellopontine angle. Facial nerve dysfunction is a debilitating phenomenon that can occur in skullbase tumors and Bell’s pals. Recovery of the facial nerve dysfunction after surgery for skullbase tumors can be disappointing, but is usually favorable in Bell’s palsy. Advances in magnetic resonance imaging (MRI) allow to visualize the facial nerve and its course in the cerebellopontine angle, also when a large tumor is present and compresses the nerve. Here, we describe the anatomical, neurochemical and clinical aspects of the facial nerve and highlight the recent progress in visualizing the facial nerve with MRI.

## Introduction

Facial nerve, the 7th cranial nerve (CN), is mainly situated in the cerebellopontine angle. Tumors arising in this area can compress this nerve. Usually, tumors do not influence the 7th CN function, but often have an effect on the 8th CN function. The 8th CN has a close relationship with the 7th CN [1]. Identification and sparing of the 7th CN during surgery is important. Therefore, it is often attempted to identify the 7th CN nerve preoperatively, but this is difficult on routine images.

The nerve is stretched around the tumor and cannot be discriminated easily from tumor tissue. The most often encountered tumors in the cerebellopontine angle are vestibular schwannomas, meningiomas, epidermoids, cholesteatoma, and metastases [2]. A skullbase tumor can cause partial or complete facial nerve dysfunction. Other presentations of facial nerve palsy, often unrelated to a mass lesion, can be infectious (Bell’s palsy), myokymia, dyskinesia, and hemifacial spasm.

Magnetic resonance imaging (MRI) is the standard diagnostic tool to visualize the cerebellopontine angle. MRI scan soft tissue well and can therefore identify the CN’s and lesions adequately [3]. MRI can visualize facial nerve originating from the brain stem and during its course in the cerebellopontine cistern, towards the internal auditory canal. Following advancing technology for MRI more imaging modalities arose, including choices in field strengths (1.5 T, 3 T, and 7 T) and options in diffusion weighed protocols that increase the accuracy and identification of anatomical structures.

In this paper, we summarize the knowledge on the anatomical details of the 7th CN at the level of its brainstem nucleus and its main branches (mainly the cisternal and internal auditory segments). Subsequently, we explore the opportunities of these new imaging modalities to visualize the 7th CN and its surroundings.

## Anatomy

**Fig. 1:**
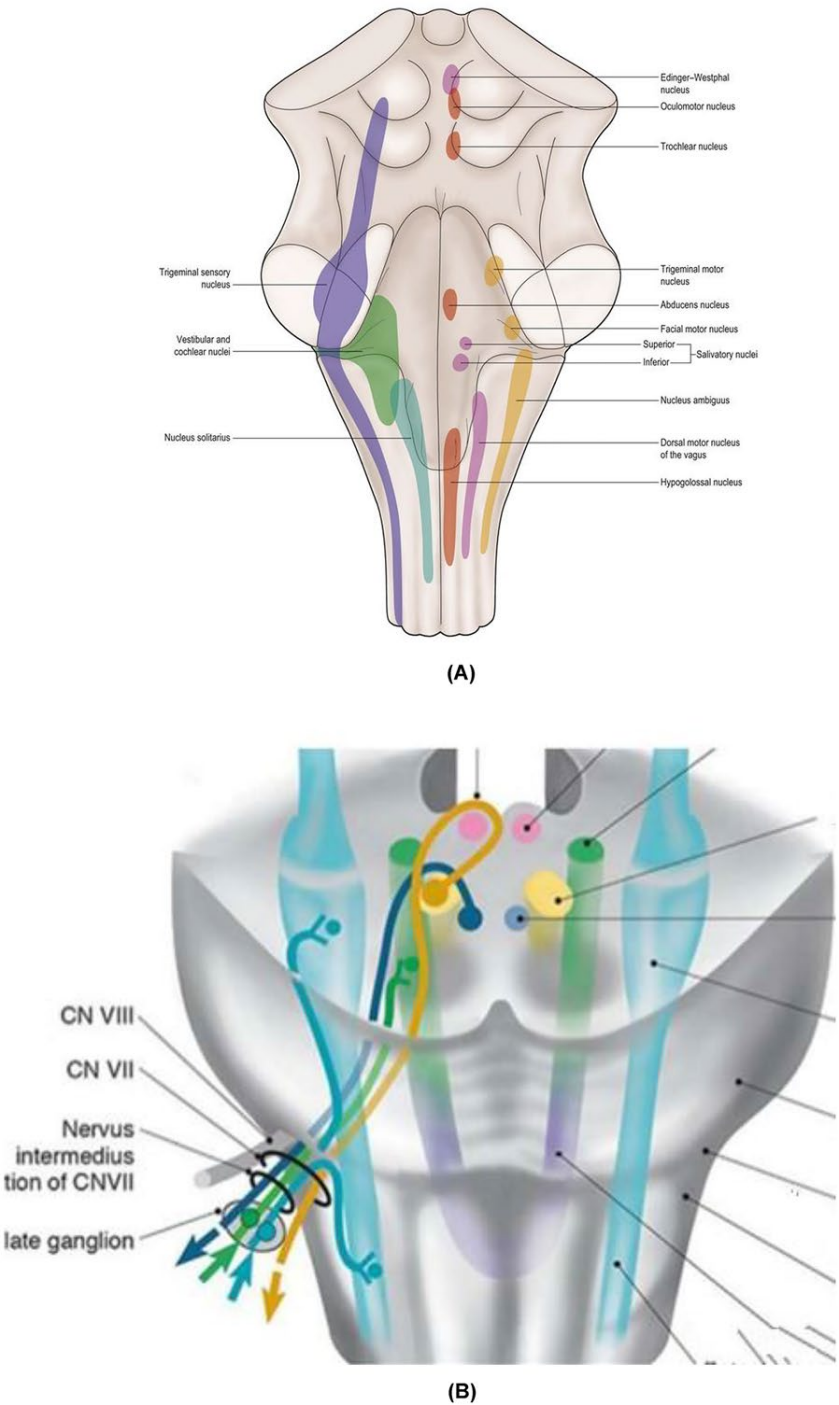
The location of nucleus with the facial nerve: the motor nucleus, the largest nucleus of facial nerve, is situated in the caudal portion of the ventrolateral pontine tegmentum. The superior salivary nucleus is located dorsal to the motor nucleus. The third nucleus, the nucleus solitarius, is located posterolateral to the motor nucleus and upper medulla

The facial nerve fibers are mixed with sensory, motor, and parasympathetic nerves (see Fig. 1). The facial nerve nucleus is one of the cranial nerve nuclei. It is located on the ventrolateral side of the lower pons' reticular structure and contains large motor cells, which are particular visceral motor nuclei. The axons emitted by this nucleus go back inward and gather into a bundle on the inside of the abducens nucleus, bypasses the ventral side and folds to the ventral side, and exits the brain along the outside of the facial nucleus. It innervates the stylohyoid muscle, the posterior abdomen of the digastric muscle, and all the expression muscles. The superior salivary nucleus is located in the pontine tegmentum of the brainstem. "General visceral motor fibers" originate from the epicrine salivary nucleus of the pons and belong to the preganglionic fibers of the parasympathetic ganglion. After the parasympathetic ganglion is replaced, the postganglionic fibers are distributed in the submandibular gland, the mucous membranes of the nose and palate, sublingual gland, and lacrimal gland. The inferior salivary nucleus is located in the upper part of the medulla oblongata. The outline of the nucleus is unclear. The neurons are scattered in the reticular structure above the dorsal vagus nucleus and the suspicious nucleus. This nucleus sends out the preganglionic fibers of the parasympathetic nerve into the glossopharyngeal nerve, through its branch petrosal nerve to the auricular ganglion for cell replacement postganglionic fibers manage the secretion of the parotid gland. The third nucleus, the solitary bundle nucleus, is located on the ventrolateral side of the dorsal nucleus of the vagus nerve. The sensory nucleus of the facial nerve, glossopharyngeal nerve, and vagus nerve control taste and general visceral sensation. The nerve fibers of the solitary tract terminate in the nucleus of the solitary tract, and the nucleus sends out fibers, part of it ascends to the diencephalon, transmitting the visceral impulse to the higher center; the other part of the fibers ends at the motor nucleus of the brainstem, completing various visceral reflex activities. It receives information from two-thirds of the tongue through general sensory and special taste signals from the auricle and the external auditory canal (Fig. 1). Nerve fibers from three facial nuclei appear on the surface of the junction of the medulla oblongata of the bridge (root appearance area [REmZ]) and adhere to the bridge about 8–10 mm before departing from the brainstem (Table 1; Fig. 1) [4].

**Table 1:** Clinical characters of facial nerve

Facial nerve	Cranial nerve	7th cranial nerve
	Fibers	Mixed with sensory, motor, and parasympathetic nerves
	MR imaging	A hypointense structure surrounded by hyperintense liquor under T2-weighted MR
Facial nerve nucleus	• Visceral motor nuclei, located on the ventrolateral side of the lower pons' reticular structure; • Innervates the stylohyoid muscle, the posterior abdomen of the digastric muscle, and all the expression muscles	
	Superior salivary nucleus	General visceral motor fibers and belong to the preganglionic fibers of the parasympathetic ganglion, located in the pontine tegmentum of the brainstem
	Inferior salivary nucleus	Located in the upper part of the medulla oblongata; Preganglionic fibers of the parasympathetic nerve
	Solitary bundle nucleus	Located on the ventrolateral side of the dorsal nucleus of the vagus nerve

### Route emerging zone

The facial nerve is composed of two roots. The larger motor root exits the brain from the pontine cerebellar angle area and the lateral part of the pontine medulla oblongata. The root enters the door of the inner ear to form a trunk, passes through the bottom of the inner ear canal, and enters the facial nerve canal adjacent to the tympanic cavity of the middle ear. There is an enlarged ganglion geniculi in the facial nerve canal. There are many branches when the facial nerve passes through the facial nerve canal and finally passes through the parotid gland. [5]. In this article, the focus is on the cisternal and intracanalicular segments.

### Cisternal segment

**Fig. 2:**
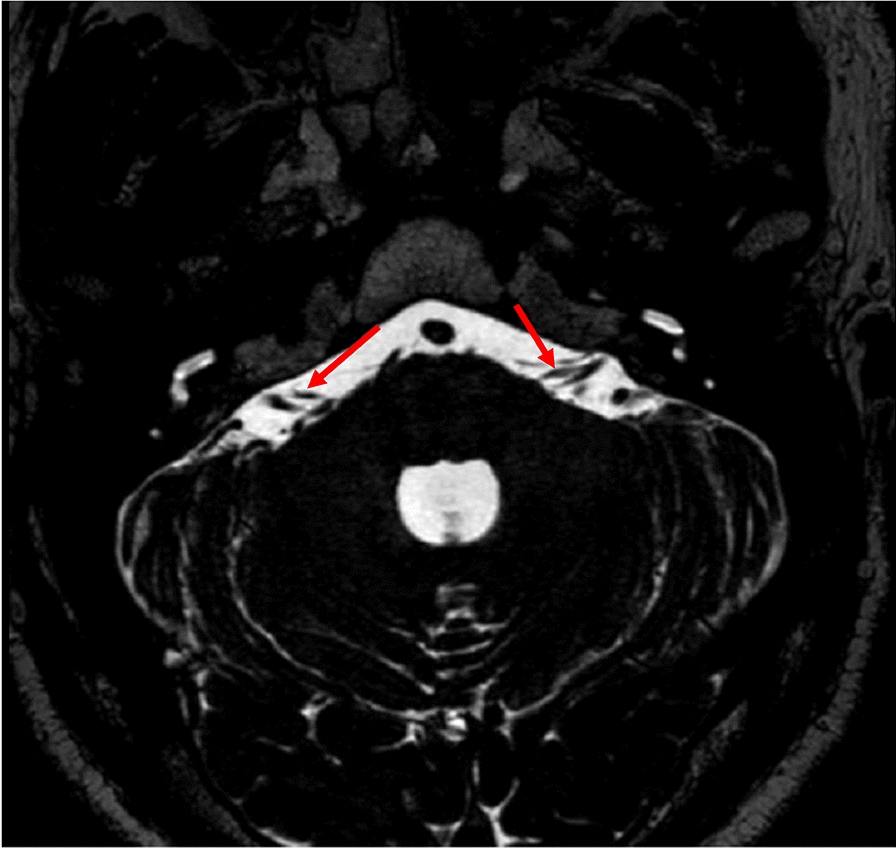
Normal MRI anatomy of cisternal segment of the facial nerve: axial T2-weighed MRI image showing the cisternal part of the facial nerve with the red arrows, anterior to CN VIII

The cisternal segment of facial nerve (FN) starts when the nerve leaves the brainstem, extends to the cerebellopontine angle (CPA) pool, and enters the ear porus of the inner auditory canal (IAC), which is about 24 mm in length [6]. It was determined to be the front of the vestibular cochlear nerve (cranial nerve VIII) in the CPA cistern by high-resolution axial T2-weighted MR sequence [7] (see Fig. 2).

### Intracanalicular segment

**Fig. 3:**
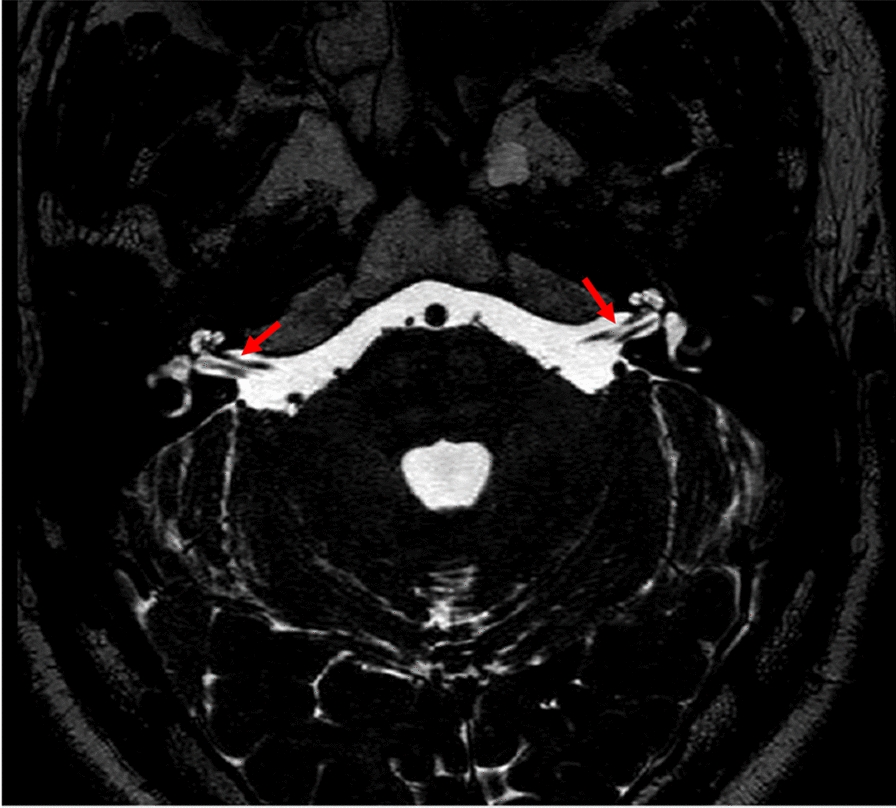
The MRI image of intracanalicular segment of the facial nerve: axial T2-weighted sequence of the IAC demonstrates the normal intracanalicular segments of the CN VII (red arrows), anterior to CN VIII

The inner section of FN extends outward from the ear porus acusticus inside the IAC and is approximately 8 mm long (Fig. 3). In addition, on the lateral side of IAC, the sickle crest separates the upper facial nerve from the cochlea and inferior vestibular nerve. The vertical crest (Bill's bar) of the variable ossified arachnoid tissue separates the anterior part of FN from the superior vestibular nerve, and the posterior part further separates the upper compartment. Oblique sagittal T2 MR could reorganize FN and the superior vestibular nerve as independent structures located above the cochlear nerve and below the inferior vestibular branch (see Fig. 2).

## Histological anatomy

**Fig. 4:**
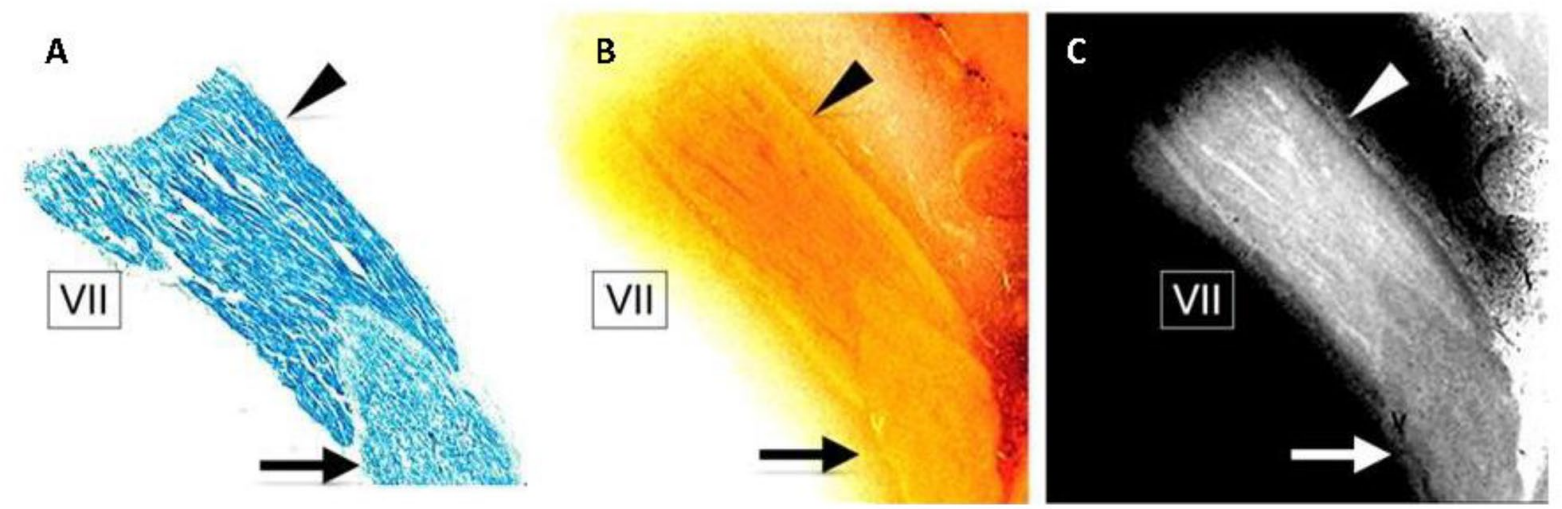
Facial nerve leaving brain stem nuclei: **a, b** The staining with Luxol fast blue (**a)** and block-face photography image (**b)** demonstrate the root of facial nerve. **c** Gray-scale image with inverted gradation of panel E with the facial nerve. This figure was adopted from the research of Keiya Iijima, etc. [4] with permission

As with other motor nuclei, the facial nucleus shows a rich acetylcholinesterase positivity with immunohistochemical stainings (Fig. 4) [8]. Studies have also shown that facial nerve fibers in the brainstem appear on the surface of the brainstem deep in the sulcus medulla of the pons and run along the surface of the pons before being separated from the pons [4].

In the same cadaver, nerve branches' number and distribution pattern show the difference between the left and right sides. The facial nerve fibers of REmZ include parallel fibers without funicular-like structures, similar to the white matter nerves in central nervous system (CNS) [4, 9]. The research of Laude and Foulon determined that the facial nerve in the pontocerebellar cistern has a fascicular organization; however, the number of nerve bundles is highly variable, and the facial nerve rotates slightly around its main axis.

## MRI anatomy

The high spatial resolution imaging of MR has become the standard examination method for facial nerve [10]. The facial nerve can be detected from the brainstem and reaches the fundus through the pontine cerebellar cistern and IAC, which is a T1- and T2-weighted sequence with high spatial resolution. Facial nerve imaging is shown as a hypointense structure surrounded by hyperintense liquor under T2-weighted MR. In addition, it has no real fascicular tissue of the facial nerve in the pons cerebellum cistern and the internal auditory canal. However, the tissue with epineurium starts from GG. In the proximal–distal mode, the number of fascicles increase and their diameters decrease. Otherwise, some nerves have a low number of fascicles (2–6) and the others have a high number of fascicles (7–15) in the extratemporal part. The number of bundle branches along the facial nerve changed, extremely the portion of extratemporal [11].

## MR anatomy of facial nerve

**Fig. 5:**
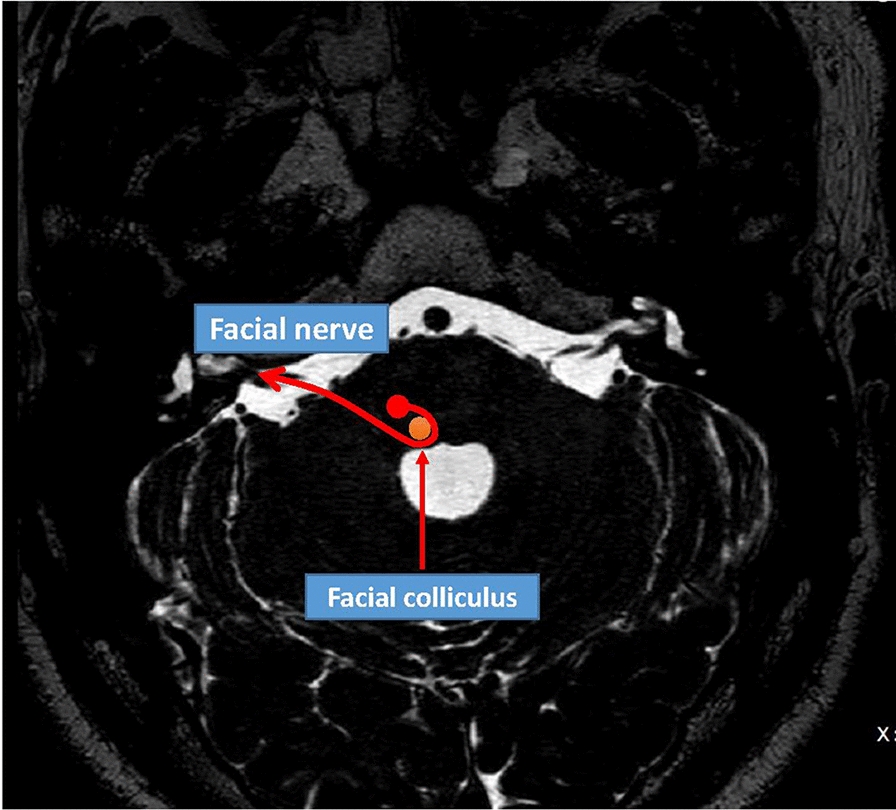
Facial colliculus of MRI: T2-weighted MRI image at the level of the facial colliculi demonstrates the course of the facial nerve within the pons. Note the route of the facial nerve with a loop around the abducens nerve nucleus (saffron one)

MR has soft tissue contrast, which can flexibly image the facial nerve. The pontine nucleus of the facial nerve cannot be depicted in an MRI scanning. However, the facial colliculus is a prominent bulge at the bottom of the fourth ventricle, which is an important marker for identifying the approximate position of the facial nerve motor nucleus (Fig. 5, facial colliculus). Facial nerve exerts as a hypointense linear structure in the high-resolution T2-weighted image, extending from the brainstem to the IAC and in front of the vestibular cochlear nerve, which is surrounded by T2 hyperintense cerebrospinal fluid. It is hard to judge the facial nerve from the three branches of the vestibular cochlear nerve in the IAC. However, because it is in front of the vestibular nerve and above the cochlear nerve, the facial nerve can be distinguished from the vestibulocochlear nerve. On the lateral side of the IAC, the 7th nerve is usually parallel to the vestibular nerve’s superior portion, and the facial nerve is hyperintense compared to the low signal in the T1-weighted image [12–14]. The oblique sagittal reformatted heavy T2-weighted MR image can distinguish the intracanalicular segment (in IAC), and using the T1-weighted sequence can best show the facial nerve along with the tympanic, labyrinthine, and mastoid segments [12, 15]. Using a microscopic coil with high-resolution T1-weighted images can best visualize the proximal extracranial portion of FN in the parotid gland.

## Facial nerve imaging in schwannomas

Skullbase tumors include meningiomas, chordomas, chondrosarcomas, esthesioneuroblastomas, pituitary tumors, parasellar tumors, vestibular schwannomas, squamous cell carcinoma, and metastases, and these tumors can be divided into malignant or benign, with some overlap [2]. In this article, we focus on FN schwannoma and vestibular schwannoma.

### Facial nerve in facial nerve schwannoma

Facial nerve schwannomas (FNS) are intracranial benign tumors originating from the Schwann cells. They can appear in any segment along the nerve sheath from the brainstem to the neuromuscular junction, but they are mainly located in geniculate ganglion (GG) [16]. The signs and symptoms of facial nerve schwannomas vary with the anatomical site of origin. The most common symptom of facial nerve schwannomas is facial paresis, combined with dizziness, pain, hearing loss, and a parotid mass and less occasionally. Imaging with a combination of MRI is critical to the initial diagnosis.

On T1-weighted images, intracranial FNS show a hypointense or isointense signal intensity compared with brain parenchyma. Extracranial FNS present a low-intermediate or intermediate signal density and are isointense to muscle on T1-weighted images. It may represent a typical “string sign” if the mass is located below the stylomastoid foramen with a beak like protrusion into it [17]. On contrast-enhanced T1 images, it manifests an enhancement of FNS and shows a typical “hourglass” appearance at the GG [18–20]. Typically, the lesions of extracranial and intracranial FNS demonstrate a homogeneous or heterogeneous hyperintense signal on T2-weighted images [20, 21].

The FNS may contain cystic foci, and the cysts present hypointensity on axial T1-weighted images and axial contrast-enhanced T1-weighted images. However, T2-weighted images show hyperintense for cystic lesions of FNS [20, 22, 23].

### Facial nerve in vestibular schwannoma

Vestibular schwannoma (VS) is the most common benign tumor arising from vestibulocochlear nerve’s (8th nerve) Schwann cells. VS represent around 80% of lesions in CPA and 8–10% of all neoplasms in the brain [24, 25]. The clinical symptoms of VS are primarily related to the oppression of the nearby tissues and cranial nerves. The clinical symptoms of VS often appear as hearing loss, tinnitus and/or disequilibrium. It also presents with facial nerve weakness, hydrocephalus and lower cranial nerve dysfunction in larger tumors [26]. Identifying and diagnosing VS with FNS is particularly difficult sometimes. The imaging technology of MRI plays an essential role in the assessment of VS lesions.

**Fig. 6:**
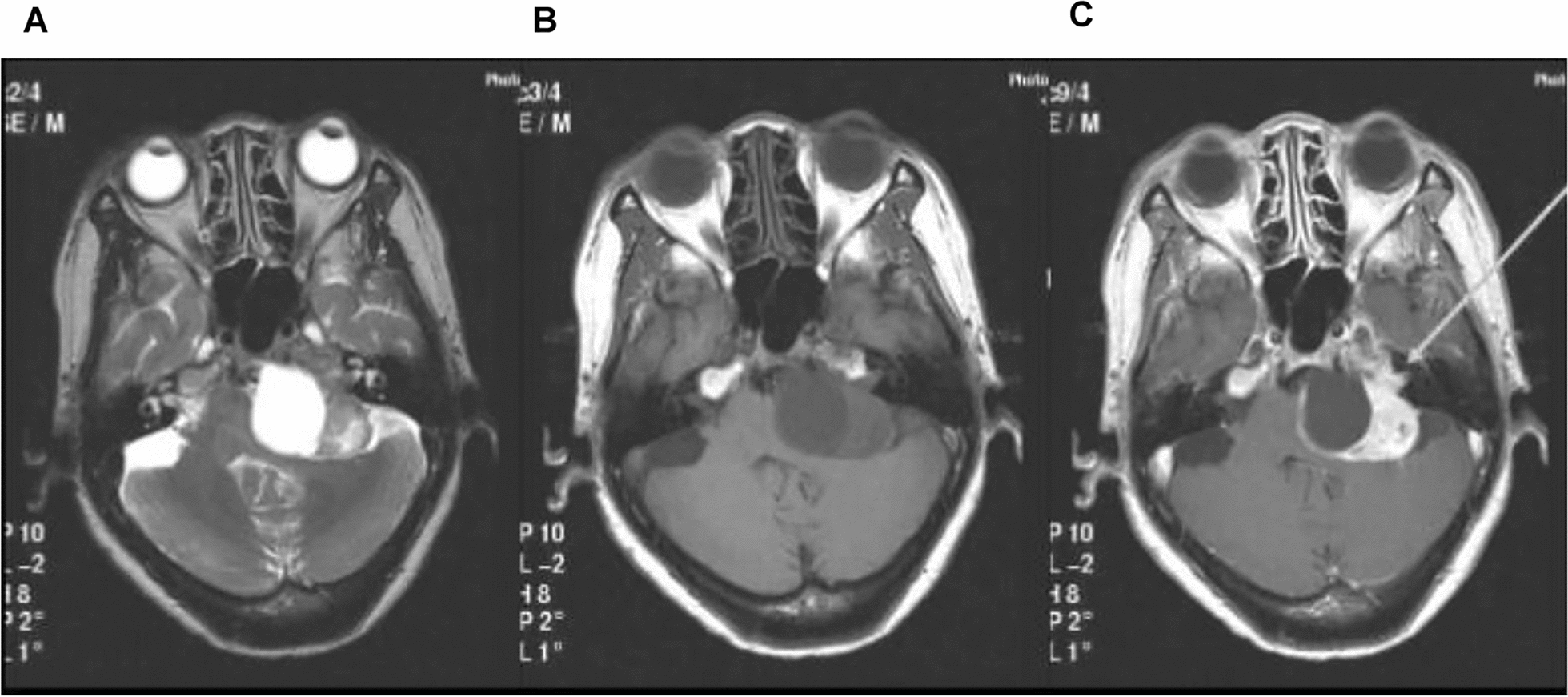
MRI scanning with VS of left cerebellopontine angle: **a** T1-weighted MRI shows the hemorrhage as an isointense area. **b** MRI depicts hemorrhage as a hypointense area on the T2-weighted images. **c** T1-weighted MRI with contrast demonstrates heterogeneous enhancement of VS

VS are typically isointense or hypo- to isointense to the brain parenchyma on T1-weighted images [27, 28]. Besides, T2-weighted axial MRI shows low hyperintense or hyperintense [27–29]. T2-weighted sequences can accurately detect the tumor size and identify the tumor growth [30]. The 3D sequence can be nicely used to assess the inner ear. For example, the thin-slice T2-weighted 3D sequence can sufficiently distinguish the origination of VS whether they are from the superior or inferior vestibular nerve [31]. The “gold standard” to diagnose or monitor VS is the enhanced T1-weighted MRI sequence with gadolinium [30]. On contrast-enhanced T1-weighted images, VS demonstrate heterogeneous or homogeneous or heterogeneous enhancement depending on the texture of the tumors [27, 30, 32, 33]. The cystic mass lesion may show a typical enhancement effect of “cyst wall” with gadolinium contrast [34]. The high-resolution T1-weighted sequence can detect very small tumors before and after the application of the contrast agent (Fig. 6).

Compared with the thin-slice T2-weighted 3D sequence, the sequence of 3D constructive interference has a higher spatial resolution and contrast-to-noise ratio in the steady state (3D-CISS) [25]. Thus, 3D-CISS is appropriate for detecting the loss of inner signal in VS patients [35]. When involved fundus of the IAC, some researchers also believed that 3D-CISS images are better than contrast-enhanced T1-weighted images to examine the features influencing the surgical outcome. So using 3D-CISS to detect the signal changes of IAC may be a helpful way to predict the recovery of hearing and balance function after VS treatment [35].

### FN in vestibular schwannoma with diffusion tensor imaging sequence

**Fig. 7:**
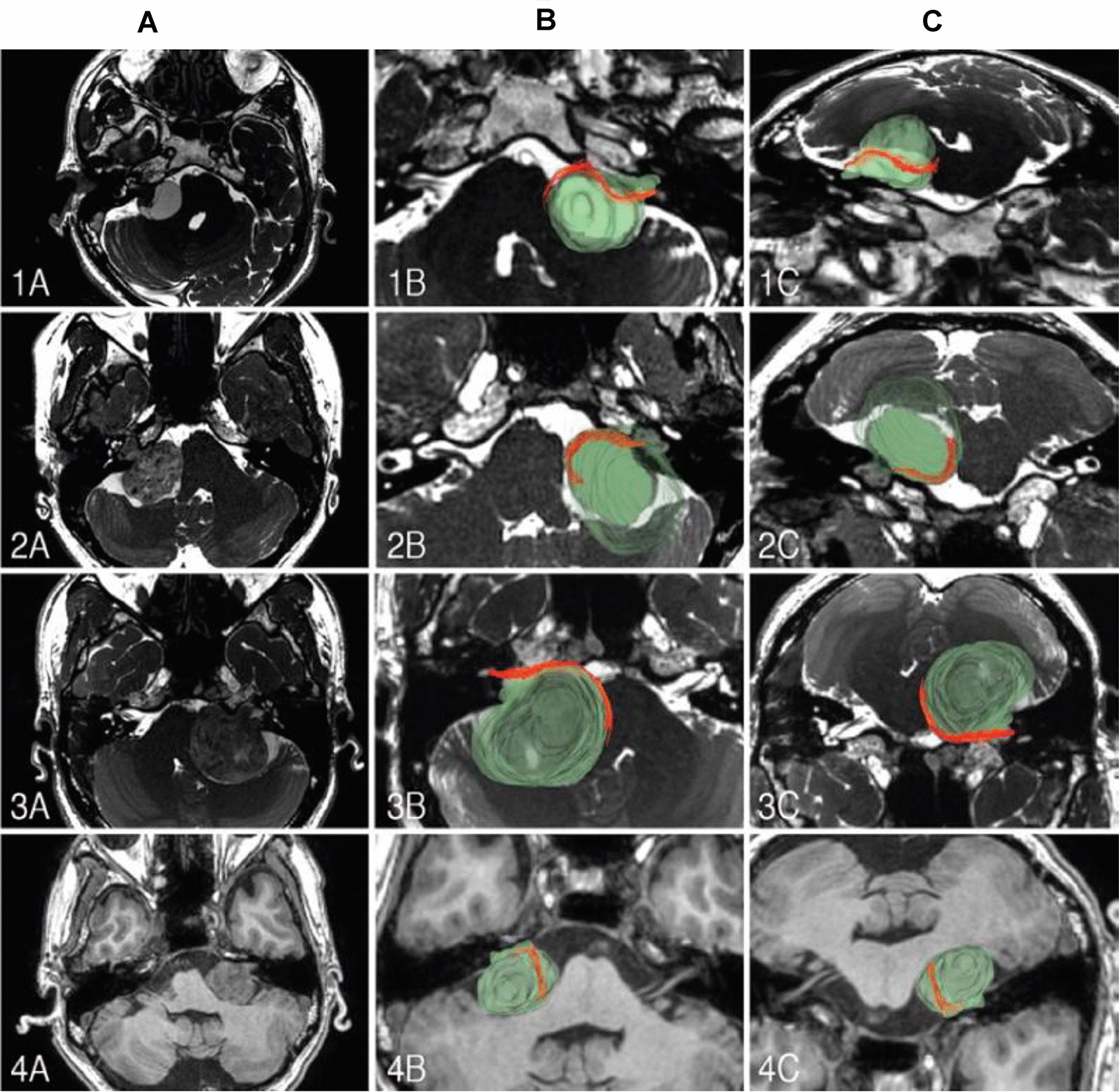
The red parts show the reconstructed FN, and green translucent parts show a 3D anatomical model of the tumor. **a** The FN is located in the anterior upper portion of the tumor; **b** the anterior lower portion of the tumor; **c** the anterior middle portion of the tumor; **d** and the anterior upper part of the tumor. This figure was adopted from the research of Fei Song, etc. [36] with permission

MRI examinations such as the 3D constructive interference in steady-state sequence and the 3D fast imaging employing steady-state acquisition (FIESTA) sequence are used to estimate FN conditions prior to acoustic neuroma surgery. Signal comparisons between nerve fibers, surrounding tissues, and CSF are used in these approaches to show neural paths [36]. However, the surrounding CSF, bone material, and tumor-related compression, on the other hand, influence the FN's imaging, making identification difficult. Diffusion tensor imaging-based fiber tracking (DTI-FT) can be utilized to precisely establish the FN's spatial position, lowering FN damage produced by electrical stimulation and minimizing injury caused by pulling. Anatomical and functional retention rates are also determined by the surgeon's ability to locate the FN's spatial position and safeguard it correctly. DTI-FT is clinically employed for intraoperative navigation due to its accuracy in tracking and reconstructing neural fasciculus in the white matter, including the 3D reconstructed pyramidal tract, arcuate fasciculus, and optic radiation [37]. Preoperative visualization of facial nerve can provide us considerable guidance before VS surgery, especially in large VS or complex VS. It has been confirmed that the use of diffusion tensor imaging (DTI) is valuable and reliable to track facial nerve fibers in pre-operation [38]. DTI has been widely applied in preoperative noninvasive visualization of white matter, but is less used in cranial nerve tracking. Recent studies showed that DTI of 3 T MRI is an effective way to track facial nerve in preoperative visualization, which is broadly consistent with the actual surgery (more than 90%). Besides, the preservation rate of facial nerve in the patients who have accepted DTI scanning proved a lot compared with the patients in whom DTI was not done [39, 40]. Thus, further studies of DTI to track cranial nerve fibers will be beneficial in skullbase tumor surgery (Fig. 7). However, because it is currently challenging to define MR parameters and perform a convenient reconstruction of FN, this application has not been widely used in clinical practice. This may be due to large fluctuations in the equipments produced by different manufacturers or the different batches made by the same manufacturer.

## Management of facial nerve dysfunction in schwannomas

### Facial nerve schwannoma

The management of FNS is still challenging. The therapeutic strategy for FNS mainly relies on tumor size and also the patients’ symptoms. Currently, the management of FNS cases includes observation, stereotactic radiosurgery (SRS) and microsurgery [18, 41–43]. Although many scholars have highly respected conservative treatment, however, some researchers recommended early surgery, although the facial and hearing functions are still well preserved before surgery in the following patients: fast-growing FNS, large FNS with temporal lobe compression, multiple-segment FNS extending in both middle cranial fossa and CPA, and intratemporal FNS broadly extending into the parotid.

For large tumors, surgical resection is the preferred treatment for FNS. Surgical therapies for FNS include total resection, near-total resection and subtotal resection [41, 44]. The purpose of surgery is to resect tumors and keep the function of FN as much as possible. When decided to treat with surgery, subtotal resection is a reasonable choice for patients with normal facial nerve function [45]. Subtotal resection can help obtain favorable facial nerve function recovery; however, some researchers indicated that it would suffer a high recurrence risk of the tumors in the long term. For total resection, it includes complete resection with grafting and total resection with preservation of FN. Nowadays, total tumor resection with nerve function preservation is technically possible. Total resection with a graft can only be executed when facial nerve function is examined House–Brackmann (HB) grade III or worse Using the HB Scale [46]. Although total resection is the current recommended treatment, however, it necessitates the sacrifice of FN segment involved in tumor and subsequent reconstruction. Near-total resection is advocated when it is difficult to dissect the tumor of FN. Long-term outcomes of FN function are favorable in patients of Near-total resection.

SRS is the application of a single high dose of radiation, stereotactically directed to an intracranial region of interest to generate or eradicate a lesion [47]. Over time, this technology has expanded to include the utilization of numerous radiation sources angled at varying angles, allowing for the construction of a range of treatment target forms. Because there is only a small dose supplied to the healthy brain, it results in long-term tumor management with only modest adverse effects when compared to whole brain radiation. SRS has been proven to be a safe and effective alternative treatment for over three decades, with excellent tumor control and a very low risk of treatment-related facial nerve morbidity [48, 49]. Given these advantages, some surgeons even recommend SRS as the primary treatment for FNS. According to the previous study, 4 FNS patients were treated with 50 Gy in 25 fractions and monitored for an average of 67.3 months. At the last follow-up, they discovered that 50% of FNS had shrunk in size, and facial nerve function in all four patients had improved or remained constant [50]. In a separate trial of 11 FNS patients treated with radiosurgery and monitored for a median of 39 months, tumor control was established in 91% of instances, and no patient's facial nerve function deteriorated [51]. As a result of these findings, patients with FNSs may benefit from early radiosurgery treatment. In patients with better facial nerve function, radiosurgery for smaller tumors resulted in a higher chance of preservation or improvement [51]. These findings showed that SRS should be the first line of treatment for patients with FNS who need treatment to save their facial function. On the other hand, Resection should be reserved for individuals who require therapy for their FNS and have a specific surgical justification.

### Vestibular schwannoma

The management for VS is to preserve better nerve function and improve life quality. The preferred management of small- or medium-sized VS includes radiotherapy, observation, and/or surgery. The management for large VS is a recommendation for surgery. Although observation for VS is a viable treatment, however, it has been reported that 31% of patients undergoing conservative management of VS needed further treatment with radiation therapy or surgery. Medical therapy like bevacizumab is also considered as an option in a clinical trial [52].

Surgical treatment is reasonable to perform for large VS; however, a surgical procedure can also be acceptable for those willing to take the risks of surgery [53]. The surgical approaches include the translabyrinthine approach (TL), middle fossa approach (MCF) and retrosigmoid approach (RS). Generally, a standard translabyrinthine approach will remove the tumor leading to profound hearing loss [33, 54, 55]. MCF and RS can provide the possibility of hearing preservation [56, 57]. Furthermore, the anatomic relationship between facial nerve and VS in each approach has a significant impact on outcomes of postoperative FN. The anterior surface of VS of FN and adhesion of the FN VS are strongly associated with worse postoperative outcomes of facial nerve. The dorsal side of the tumor indicates achievable functional preservation of FN during surgery [57]. Direct electrical stimulation can map FN in the surgical field [58]. Placement of an intraoperative continuous facial nerve stimulating electrode is helpful for the preservation of FN function.

### Management of facial nerve in VS resection

VS resection aims to resect the tumor altogether and preserve FN function. According to the analyses, the preserved function of the postoperative FN was related to the tumor size, the tumor nerve of origin, and the extent of resection [59, 60]. The tumor growth in IAC will cause the displacement and deformation of the 8th nerve, leading to the expansion of the IAC. Further development of the tumor will eventually protrude into the CPA with less resistance, which will compress the brain stem and cerebellum and produce related symptoms. Therefore, the operation of the 8th nerve involves most cranial nerves, blood vessels, cerebellum and brain stem of CPA. The FN course was divided into four patterns according to its position (Fig. 1): anterior-superior or ventral-cranial (AS), the anterior (ventral) surface of the tumor (A), dorsal, and (D) anterior-inferior or ventral-inferior (AI). AS pattern was most common (68.4%) for tumors < 1.5 cm. A and AI positions increased (separately 31.4% and 25.5%) for tumors > 1.5 cm [61]. Accurate anatomic position identification and FN course are essential to preserving the FN in vestibular schwannoma surgery. In modern-day VS surgery, MRI and neuro-electrophysiological monitoring can assist the surgeons in achieving this goal.

After making a small incision in the dura and arachnoid, the cerebrospinal fluid of the cerebellomedullary cistern was released. When the tension of the cerebellum was decreased, the surgeon needed to make a sharp separation of the arachnoid along the lateral cerebellar hemisphere to the cerebellopontine angle and the posterior cerebral nerve, petrosal vein and trigeminal nerve were carefully preserved. The capsule of the posterior wall of the tumor was cut off, and the tumor was removed in situ with ultrasonic suction and a laser knife. After decompression in the tumor, the tumor capsule wall was separated and removed in turn. Most of the tumors in the internal acoustic meatus could be removed using the arachnoid space. Some of them can only be removed after grinding the posterior lip of the internal auditory meatus for the limitation of operation space; finally, dissect the residual tumor adhering to the facial nerve.

The location and shape of the facial nerve and cochlear nerve in the cisternal segment are changeable because of the giant acoustic neuroma's different origins and the tumor's compression. Although the nerves have a rotation while entering the internal acoustic meatus, the bone ridge separation makes the position of the nerves relatively constant and easy to identify. Therefore, the operation in internal acoustic meatus is recognized as the key to judging and protecting the facial nerve during acoustic neuroma surgery. Chinese people's inner ear hilum's anterior–posterior diameter is (10.36 ± 2.56) mm, the upper and lower diameter is (4.62 ± 0.83) mm, the length of the inner ear canal is 10 mm, and the transverse ridge is 6.84 mm. Therefore, the lip should not exceed 1 cm when grinding the internal acoustic meatus to avoid the wreck of the bone labyrinth. The facial nerve in the internal acoustic meatus is always so severely squeezed that it is difficult to distinguish it from the arachnoid. Therefore, in this case, electrical stimulation can be a reliable method to judge the FN.

#### Electrophysiological monitoring

**Fig. 8:**
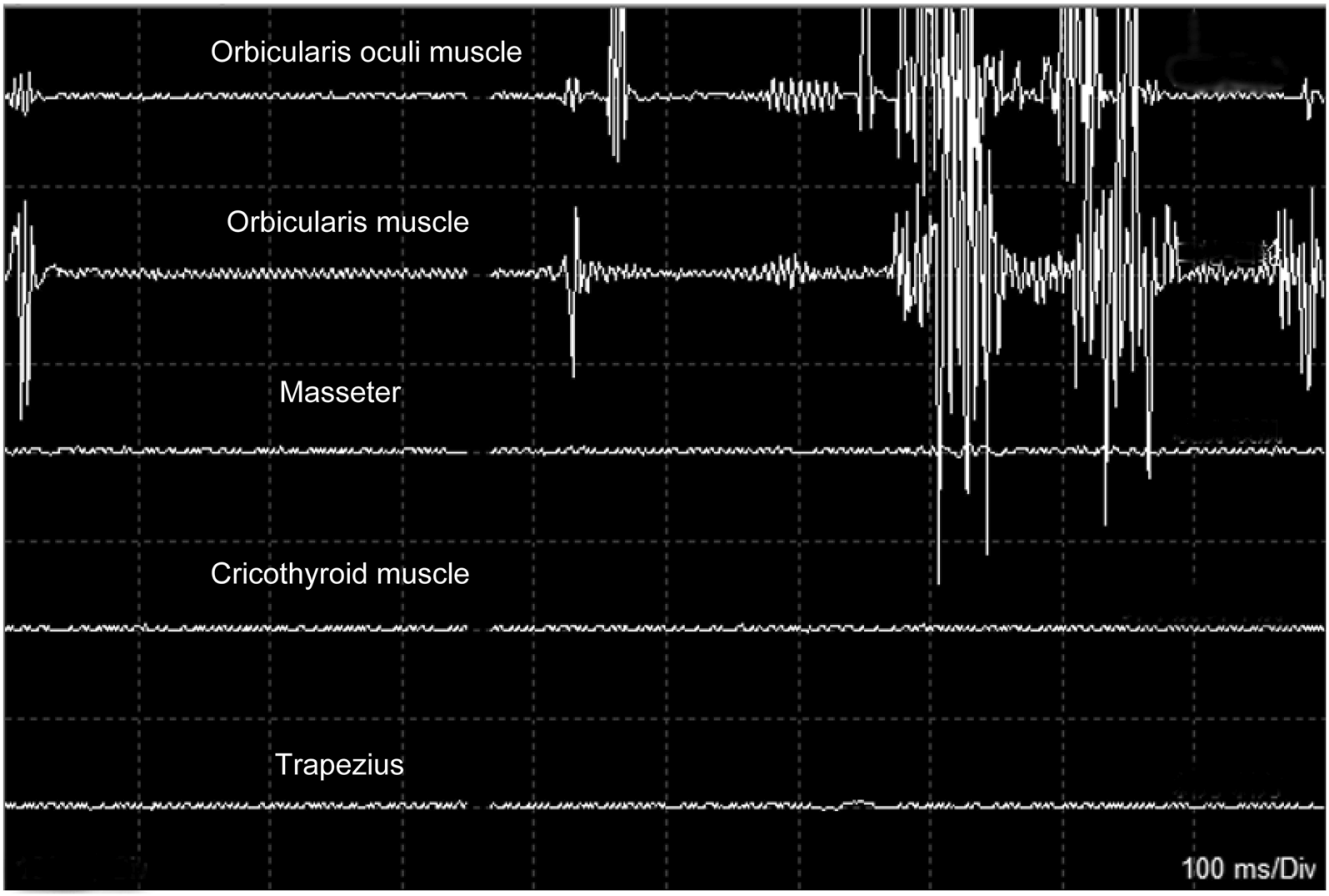
EMG in FN monitoring: when the facial nerve is harassed, the electrodes record the potential of the orbicularis oculi muscle and orbicularis oris muscle

The introduction of electrophysiological monitoring to VS surgery has significantly improved the rate of total tumor removal, FN anatomical preservation, and postoperative FN function. The FN monitoring technique includes the free-running electromyography (EMG), direct stimulation of the FN and facial motor-evoked potential (FMEP) [62]. In free-running EMG, electrodes were inserted in the orbicularis oculi muscles and orbicularis oris, providing timely electrical signal feedback to the surgeons when irritating the FN. With the direct stimulation of the FN, surgeons can identify the FN more accurately [63]. However, EMG has a limitation; the prognostic role in facial nerve outcomes remains less evident. In patients undergoing skullbase surgery, FMEP has recently been regarded as a standard intraoperative neurophysiological method in predicting FN function postoperatively. FN function immediately after surgery was significantly correlated with the orbicularis oculi muscle FMEP ratio, which was 80% (*P* = 0.037) and 35% (*P* = 0.000). The FMEP loss is tightly linked to postoperative facial paralysis, albeit to varying degrees. The combination of electrophysiological monitoring can help preserve the FN (Fig. 8).

#### MRI

Fiber tracking based on diffusion tensor imaging is a mature imaging technology that can perform 3D reconstruction of white matter fibers, an accurate method for preoperatively identifying the facial nerve concerning vestibular schwannomas. FN preoperative visualization using DTI-FT was observed in 96.6% of patients, while 90.6% of visualization were consistent with the surgery [36, 64].

#### Electrophysiological monitoring

Compared to simple microsurgery, electrophysiological monitoring-assisted microsurgery can effectively improve the rate of total tumor resection [65], the intraoperative anatomical retention rate of FN, postoperative protective rate of nerve function, and postoperative symptoms of facial paralysis [63]. Neurophysiologic monitoring of FN was first introduced into the operating theater in the 1970s, which included transcranial electrical stimulation-induced FMEP and EMG.

## Discussion

The FN route is very complicated, and lesions from this pathway can primarily affect its function. Schwannomas, which generally occur in CPA, are the common cause for this pathogenesis. MRI is the preferred medical imaging technique that can be employed to clarify the diagnosis and locate the lesion site. Understanding the MRI anatomy of facial nerve is very important for radiologists and neurosurgeons to diagnose and manage underlying pathology. The goal of the management for schwannomas is to preserve or improve neurological function and remove the compression of these tumors. Generally, treatment recommendation for medium- or small-sized schwannomas include observation, radiotherapy, and/or surgery, and for large schwannomas, surgery is advocated. Preoperative identification of the 7th CN during surgery is necessary but is also difficult. Currently, the DTI sequence of MRI provides a possibility to track the facial nerve fibers preoperatively. However, the effect of reconstruction of facial nerve with imaging technology needs to be further confirmed because the efficiency of reconstruction is not very high, and it is also necessary to distinguish the facial nerve from the vestibular nerve. Likewise, a consensus concerning DTI parameters for fiber tracking of facial nerve should also be required to consolidate.

## Conclusion

In conclusion, the entire management of the facial nerve should always be included in the treatment strategy for a patient with parotid malignancy. The available diagnostic procedures with imaging technology could be used to determine the tumor's relationship to the nerve, as well as where dissecting and preserving the nerve may be challenging. The primary goal is to completely remove the tumors while preserving the facial nerve if possible. In the decision-making process, the patient's age, risk of facial function loss, hearing loss, and ongoing growth are all crucial factors to consider. Total resection with nerve grafting should be undertaken for patients with moderate to severe facial paralysis if preservation of the facial nerve is not possible, but subtotal surgical resection is appropriate for those with good face function. Clinicians are hoping to have a better knowledge of single-modality treatment failures so that they may better counsel patients and guide treatment.

## Data Availability

All data generated or analyzed during this study are included in the present article.

## Abbreviations

CN: Cranial nerve
MRI: Magnetic resonance imaging
CPA: Cerebellopontine angle
IAC: Inner auditory canal
FN: Facial nerve
CNS: Central nervous system
FNS: Facial nerve schwannomas
GG: Geniculate ganglion
VS: Vestibular schwannoma
CISS: Contrast-to-noise ratio in the steady state
FIESTA: Fast imaging employing steady-state acquisition
DTI-FT: Diffusion tensor imaging-based fiber tracking
SRS: Stereotactic radiosurgery
HB: House–Brackmann
TL: Translabyrinthine approach
MCF: Middle fossa approach
RS: Retrosigmoid approach
AS: Anterior-superior or ventral-cranial
EMG: Free-running electromyography
FMEP: Facial motor-evoked potential
